# Growth characteristics and classification systems of hemifacial microsomia: a literature review

**DOI:** 10.1186/s40902-024-00427-8

**Published:** 2024-05-11

**Authors:** Joonyoung Huh, Ji-Song Park, Buyanbileg Sodnom-Ish, Hoon Joo Yang

**Affiliations:** Department of Oral and Maxillofacial Surgery, School of Dentistry, Seoul National University, 101, Daehak-Ro, Jongno-Gu, Seoul, 03080 Korea

**Keywords:** Hemifacial microsomia, Growth, Deformity, Classification

## Abstract

**Background:**

Hemifacial microsomia is characterized by the hypoplasia of the mandible and temporomandibular joint, involving a variety of abnormalities of the craniofacial area. Since it gradually worsens as patients grow, it is necessary to understand the characteristics of facial bone growth and facial deformity in hemifacial microsomia patients in order to determine appropriate treatment timing and treatment methods.

**Main body:**

Appropriate classification of hemifacial microsomia would facilitate accurate diagnosis, selection of treatment methods, and prognosis prediction. Therefore, in this article, we review previously published hemifacial microsomia classification and provide an overview of the growth of the facial skeleton and the characteristics of hemifacial microsomia-related facial deformities. The OMENS system is the most comprehensive classification method based on the characteristics of hemifacial microsomia deformity, but it needs to be improved to include malar/midface abnormalities and nerve involvement. In hemifacial microsomia, growth is progressing on the affected side, but to a lesser degree than the unaffected side. Therefore, surgical intervention in growing patients should be performed selectively according to the severity of deformity.

**Conclusion:**

Understanding growth patterns is important to develop appropriate treatment protocols for correcting asymmetry in adult patients and to minimize secondary anomalies in growing patients.

## Background

Hemifacial microsomia (HFM) is the second most common craniofacial abnormality after cleft lip and palate, with an estimated frequency of approximately 1 in 3,500 to 6,000 live births [1–3]. HFM has a different phenotype, and has several names, such as hemignathia, otomandibular dysostosis, lateral facial dysplasia, auriculobranchiogenic dysplasia, and microtia syndrome [2]. Since HFM contains the structures of the first and second pharyngeal arches, it mainly includes hypoplasia of the unilateral condyle and ramus, and a very diverse abnormalities of the maxilla, facial nerve and trigeminal nerve, external and middle ear, masticatory muscles, and soft tissue [2–4].

The treatment protocol for patients with HFM is determined by the specific facial deformity exhibited by the patient. Surgical treatment to correct facial asymmetry for growing patients with HFM mainly seek to increase the mandibular dimension by performing distraction osteogenesis (DO), or to reconstruct the mandibular condyle with growth potential by performing a costochondral graft [5–10]. After growth is complete, orthognathic surgery with/without temporomandibular joint (TMJ) reconstruction can be performed according to the facial deformity [5, 10, 11]. In order to select an appropriate treatment method, it is important to understand both the growth pattern and the facial deformity characteristics. If the surgeon recognizes the growth pattern of the HFM for each part of the mandible, the treatment method can be focused on the area where growth is insufficient, to achieve dimensions and angulation similar to the unaffected side [4].

In order to accurately diagnose HFM patients, an appropriate classification method is needed. HFM, for example, can often be confused with hemimandibular hypoplasia. In contrast to the tendency toward asymmetry recurrence on long-term follow-up after early interventions such as DO in growing children with HFM, some reports described surprising postoperative stability despite a similar phenotype [12, 13]. These patients may have been misdiagnosed with HFM when they had isolated hemimandibular hypoplasia (pseudo-HFM). Unlike HFM, hemimandibular hypoplasia is not diagnosed at birth; is not associated with soft tissue defects, ear defects, or nerve deficits; and the masseter muscles are well developed. Also, unlike HFM where the affected side is flat, fullness is observed in the affected side of hemimandibular hypoplasia. The radiological findings of hemimandibular hypoplasia are very specific, and include hypoplasia of the condylar and coronoid processes and ramus, which typically collapse on one another, and a typically V-shaped sigmoid notch [12, 13]. Since Pruzansky first classified and reported the severity of the mandibular and TMJ deformities seen in HFM [14], various classifications of disease/deformity of HFM have been suggested, that have informed diagnosis and treatment methods [8, 15–28]. However, a generally accepted classification of HFM has not yet been established.

Therefore, the purposes of this review are to provide an overview of the features of growth of the facial skeleton and facial deformity characteristics in HFM patients, and to summarize previously published HFM classification systems.

## Main text

### Growth and facial deformities in hemifacial microsomia patients

In HFM, the affected side does not grow in proportion to the unaffected side [29–31]. This asymmetry of the jaw may not be evident in infancy due to the abundant buccal fat pad, but becomes more pronounced in the middle of the first decade or in puberty, when mandibular growth increases [2]. The unaffected side continues to grow, but it doesn`t show more overgrowth compared to the growth of the normal mandible [4, 30].

In the mild type, Kaban type I, the mandible shows a thinner condylar cartilage in slightly hypoplastic mandibular condyle (Table 1). However, hypertrophy of the chondrocytes and endochondral ossification are quite normal. Therefore, mandibular growth of Kaban type I is expected to be only slightly deficient. On the other hand, the severe type of HFM, Kaban type III, is associated with aplasia or severe hypoplasia of the mandibular cartilage. The mandibular condyle lacks condylar cartilage and endochondral ossification, and the mandibular growth on the affected side may stop prematurely [3].

**Table 1:** Pruzansky-Kaban classification [8]

Type	Malformations
Type I	Small mandible with normal shape
Type IIA	Hypoplastic mandible with abnormally shape/ Glenoid fossa in normal position
Type IIB	Abnormally shaped mandible/ TMJ displaced forward, inward, or downward
Type III	Complete loss of the ramus and TMJ

As a clinical symptom of HFM, congenital facial nerve palsy may occur. Although facial muscles, which are innervated by the facial nerve, are important for craniofacial growth and development, the occurrence of facial nerve palsy in HFM does not affect growth of the maxilla and mandible. However, HFM patients with facial nerve palsy tend to show less consistent asymmetry in the maxilla and mandible. Choi et al. [32]. explained that subtle changes occurring in the midfacial bones and mandible due to facial nerve palsy may result in the lack of correlation between the maxilla and mandible.

#### Mandibular growth pattern and resultant mandibular deformities

The growth curve of the mandibular ramus in HFM patients (Pruzansky’s type I—III) is similar to that of normal control group. Therefore, HFM patients have smaller mandibles than normal even when they are young, as well as after growth [33]. Meazzini et al. reported that the ratio of mandibular ramal height between affected and unaffected sides was 57 ± 15% at an average age of 5.9 years in Pruzansky’s type I and II patients, and that these patients showed a ratio of 58 ± 15% even when growth was complete. This means that growth is progressing on the affected side, but to a lesser degree than the unaffected side [34]. Solen et al. analyzed the growth of 9 patients with Pruzansky's type I and IIA HFM from 12.4 to 14.4 years of age, divided into condyle and posterior ramus. The annual growth rate of the condyle was 3.1% lower on the affected side than on the unaffected side, and this was not statistically significant. On the other hand, the posterior ramus grew at a rate of 2.6 ± 1.7 mm/year on the affected side and 3.6 ± 2.4 mm/year on the unaffected side, showing a statistically significant difference of 33.5% [35]. HFM patients also showed mandible widening, and the mean annual change was reported to be 0.07 mm [36]. However, this was less than the mean annual change of 0.13 mm reported in normal patients [37].

Kim et al. divided the mandible into condyle, coronoid, body, and angular units, and measured the size and angulation of each unit in children, adolescents, and adults with HFM. The measurements were compared between the unaffected side and the affected side, as well as between HFM patients and a normal control group. The condylar and coronoid units of the affected side in Pruzansky’s type II increased with age, as opposed to the angular and body units. The body and condylar units in Pruzansky’s type II and III showed a tendency to decrease the angle between the affected side and the unaffected side with age, unlike the coronoid and angular units [4].

HFM shows a typical feature of unilateral mandibular hypoplasia, which varies from mild to severe. HFM patients have more retruded mandibles in relation to the maxilla. In addition, the mandibular angle shows a steeper configuration for both the affected and unaffected sides [38]. Mandible defects can range from hypoplasia to the absence of the glenoid fossa, condyle, or mandibular ramus. The chin is displaced to the affected side, because its ramus is shorter in height and mandibular body is shorter in length. Tokura et al. reported that the ratios of the affected side to the unaffected side of the transverse distance of the mandibular condyle, mandibular ramus height, and body length of the mandible were significantly lower in the HFM group than in the control group. As the chin deviates toward the affected side, the inclination of the body, the angle between the line from menton to antegonial notch and horizontal line, was significantly greater on the affected side than on the unaffected side. The inclination of the mandibular body was significantly correlated with the shift of the menton [39]. In addition, there is usually a mandibular occlusal plane cant located higher on the affected side. The mandibular body on the affected side also becomes smaller in the horizontal direction because of decreased bone deposit on the buccal surface and resorption on the lingual surface [2, 29, 33]. Although condyle/ramus complex hypoplasia was observed, approximately 14% of HFM patients showed compensatory growth of the mandibular body on the affected side [40]. Among adult patients with Pruzansky’s type II disease, when grown without treatment, the ramus length, body length, and ramus volume of the affected side are only 65.99%, 88.26%, and 52.21% of the unaffected side [41].

When evaluating the size of the mandible by dividing the mandible by region, the discrepancy in the size of the condylar unit between the affected and the unaffected side reached 6.7–10.9 mm for Pruzansky’s type II and exceeded 20 mm for Pruzansky’s type III cases. Similarly, the size discrepancies of coronoid and body units were 1.5–5 mm and 1.4–11.0 mm for type II, and 17.0–25.0 mm and 14.2–16.3 mm for type III, respectively [4].

Since unilateral or bilateral retrusion of the mandible is the most common skeletal deformity in HFM patients, there have been concerns about airway disorder. The prevalence of obstructive sleep apnea in HFM patients has been reported to be 17.6–24%, which is significantly higher when mandibular hypoplasia is severe or bilateral [42, 43]. In addition, Cohen et al. [43] suggested severe soft tissue hypoplasia, severe orbital abnormalities, abnormalities of the mandibular branch of the facial nerve, the glossopharyngeal nerve, and the hypoglossal nerve, and loss of bilateral healing as possible contributing factors to the occurrence of obstructive sleep apnea in HFM.

Protocols for how and when to treat HFM vary from surgeon to surgeon, and this is mainly correlated to their views on growth potential [44]. Those who advocate for early surgical intervention suggest that early intervention promotes growth and reduces malocclusion [5, 11]. On the other hand, those who advocate for delayed intervention are concerned about the growth impairment caused by early intervention and focus on the growth potential of HFM [6, 9, 33, 35, 41]. In addition, in long-term evaluation of DO, vertical bone growth was limited, the growth ratio of the affected side was reduced, and relapse could occur, which may be the basis of arguing for delayed intervention [6, 7, 9, 34].

### Maxillary growth pattern and resultant maxillary deformities

The maxilla grows downward and forward following mandibular growth. Mandibular growth is limited and the upper and lower teeth are occluded in HFM patients; the vertical growth of the midface is also reduced [29]. Kearns et al. reported that the angles of the piriform, maxillary occlusion, and intergonial cants increased with time in both mild (types I/IIA) and more severe forms (types IIB/III) of HFM. Overall end-stage facial anomaly was found to be significantly associated with the severity of mandibular anomaly [45]. The piriform apertures and maxillary occlusal plane were also gradually tilted upward on the shorter side, parallel to the occlusal plane of the mandible. Because the maxilla does not exhibit normal vertical growth, the piriform aperture and maxillary alveolus are not usually separated from the orbit. In addition, since the mandible exhibits unilateral undergrowth from birth in HFM patients, the vertical asymmetry of the midface involves the nose and inferior orbital rim. Therefore, the orbit may be displaced downward in HFM patients [29].

### Deformities of the skull and orbit

In HFM patients, the skull base showed asymmetry depending on the site. The most asymmetric and growth-restricted areas are the glenoid fossa and the mastoid process [46]. In contrast, there is minimal to no deviation of the anterior cranial base angle as well as absent or minor asymmetry of the endocranium [47]. These results imply that although the skull base is closed to the mandible and midface, it seems to be relatively spared from alterations in growth of the facial bones [47], and asymmetry of the skull base concentrated in the mastoid process and glenoid fossa affect facial asymmetry in HFM [46].

The OMENS classification system classifies the severity of HFM depending on orbit, mandible, ear, nerve, and soft tissue deformities (Table 2), as these 5 areas are thought to be most affected by HFM [25]. According to the study by Gribova et al. which compared the orbital volume in 39 HFM patients with 3-dimensional computed tomography (CT), orbital volume was significantly smaller by 10 ± 41% on the affected side. The affected side was smaller than the unaffected side in 80% of the sample [48]. When the orbits were evaluated clinically, 4–12% of patients were noted to have small orbits. In addition, the height of both orbits may vary in HFM patients [25, 49]. Vento et al. reported that orbital position and size abnormalities were related to the severity of mandibular hypoplasia [25]. On the other hand, other studies have not found a correlation between deformities [48, 49].

**Table 2:** OMENS classification [25]

Parts	Grades	Malformations
Orbit (O)	O0 O1 O2 O3	Normal Abnormal size Abnormal position Abnormal size/position
Mandible (M)	M0 M1 M2 M3	Normal Small mandible Hypoplastic mandible with abnormally shape/ Glenoid fossa in normal position Complete loss of the ramus and TMJ
Ear (E)	E0 E1 E2 E3	Normal Mild hypoplasia with all structures Loss of an external canal/ hypoplastic concha Displaced lobule with absent auricle
Nerve (N)	N0 N1 N2 N3	Normal Upper facial nerve affected Lower facial nerve affected All facial nerve branches affected
Soft tissue (S)	S0 S1 S2 S3	Deficiency: not obvious Deficiency: minimal Deficiency: moderate Deficiency: severe

### Dental development and occlusion

Ongkosuwito et al. compared dental developmental scores between the affected and unaffected sides in HFM patients and compared these data with those collected from normal children. They found there was no significant difference in the development of teeth between the affected and unaffected sides, which indicates that HFM patients did not have an unbalanced progression of teeth development. When comparing the dental development of both affected and unaffected sides according to the severity of HFM, patients with Pruzansky-Kaban’s types IIB and III showed significantly delayed tooth development compared with patients with types I and IIA and normal children [50]. In addition, the prevalence of missing teeth increased with increasing severity of mandibular deformity. Kaban’s type I, II, and III patients exhibited a prevalence of missing teeth of 22.58%, 23.81%, and 69.23%, respectively [51].

Yang et al. reported that 93.2% of HFM patients had angle class I and II molar relationships, and that the remaining 6.8% had class III molar relationships [40]. The inclination of the maxillary incisors was significantly smaller, and the inclination of the mandibular incisors was significantly greater than that of the normal control [38].

Telich-Tarriba et al. reported that the bite force was not decreased on the affected side compared with the unaffected side or normal controls. However, during maximum intercuspation, surface electromyography of the masseter muscle on the affected side was significantly reduced compared to the unaffected side and the control group. Hence changes in the amplitude or density of the electromyographic signals do not change the strength in a directly proportional manner [52].

### Classification of hemifacial microsomia

A classification system should aid in diagnosis of a condition, improve communication among clinicians, and help predict progression of disease/deformity [8]. The optimal categorization for any disorder is one that is easily performed, reproducible among evaluators, and helpful in predicting treatment and prognosis [53]. Many clinicians have attempted to classify HFM from different aspects; however, there is still no optimal classification of HFM that is universally accepted.

### Classifications of mandibular hypoplasia

As the first report of skeletal classification, Pruzansky suggested 3 types of mandibular hypoplastic malformation focused on the ascending ramus, condyle and glenoid fossa of the temporal bone in the late 1960s [14]. Later, type II was modified for surgical planning, with subclassification in terms of TMJ anatomy and function by Kaban and coauthors [8]. Types I and IIA have sufficient bone and adequate TMJ for DO or osteotomy, and usually do not require bone grafts. Types IIB and III require construction of the ramus/condyle units, and in some cases, the TMJ (Table 1). The main disadvantage of this classification system is a failure to address other abnormalities frequently seen in HFM patients [23].

Similar to Pruzansky’s classification, Swanson and Murray introduced 3 types of skeletal defects in HFM in terms of the mandible and TMJ as a key reference [27]. Harvold and coauthors suggested 5 subgroups of mandibular hypoplasia with masticatory muscle function [26]. In skeletal classification for surgical planning by Lauritzen et al., the zygoma arch and orbit were included in addition to the mandible and TMJ. The condyle in Type II exists with deformations in size and shape in Pruzansky’s and Swanson and Murray’s classification, while it is missing in the classification by Lauritzen et al. [20].

Recently, skeletal malformation was evaluated with high-resolution 3-dimensional computed tomographic imaging instead of conventional 2-dimensional plain radiography and clinical evaluation [19, 53]. Huisinga-Fischer et al. reported a skeletal scoring system consisting of a mandibular deformity scoring system for mandibular hypoplasia and a cranial deformity scoring system for hypoplasia of other facial bones [19]. Combining these 2 skeletal scores resulted in a comprehensive craniofacial deformity scoring system with a single numeric value. Because it does not aid in formulating surgical plans, it has not been widely adopted thus far [54]. Another study by Wink et al. compared clinical Pruzansky-Kaban scores based on clinical examination by single surgeon at the time of initial clinical presentation to a score based on CT by evaluators from a craniofacial surgery society, and to consensus ‘in-house’ scores by craniofacial surgeons. They demonstrated that there was wide variability among experts in the field regarding their interpretation and implementation of the Pruzansky-Kaban classification system. The mean evaluator agreement between the clinical Pruzansky-Kaban scores and the scores based on CT was 39.17 ± 8.83%, while that between the scores based on CT and the ‘in-house’ scores was 69.71 ± 9.42% [53].

### Classification of ear malformations

Prior to the skeletal classification report by Pruzansky [14], Meurman [22] introduced 3 classifications of external ear malformation, which range from mild loss of the auricular structure to near complete auricular aplasia (Table 3). In the first report of skeletal classification by Pruzansky [14], preauricular malformation was also graded using the modified Meurman’s system to find correlations among severity of the deformities of the external ear, temporal bone and mandible [16]. Later, Pruzansky and colleagues introduced 9 deformity combinations of the mandible and external ear (Table 4) [55].

**Table 3:** Auricle classification by Meurman [22]

Grade	Malformations
Grade I	Small, malformed auricle retaining characteristic features
Grade II	Rudimentary auricle with a hook
Grad III	Malformed lobule with rest of pinna absent

**Table 4:** 9 deformity combinations of the mandible and external ear by Pruzansky [55]

	Mandible			
Auricle		Grade I	Grade II	Grade III
	Grade I	Small, malformed auricle Small mandible	Small, malformed auricle Malformed structures	Small, malformed auricle Severely malformed ramus
	Grade II	Rudimentary auricle Small mandible	Rudimentary auricle Malformed structures	Rudimentary auricle Severely malformed ramus
	Grade III	Malformed lobule only Small mandible	Malformed lobule only Malformed structures	Malformed lobule only Severely malformed ramus

In the report by Longacre et al. in 1965, 44 HFM patients were divided into 2 groups: unilateral or bilateral facial microtia for the purposes of treatment planning. These 2 groups were further subdivided into 4 classes of increasing facial deformity (Table 5) [21]. However, the microtia was not graded, nor was facial deformity clearly defined [16, 53].

**Table 5:** Classification of microtia by Longacre et al. [21]

Type	Treatment
Unilateral/Bilateral microtia with - No/slight deformity of the face - Moderate/severe deformity	Otoplasty only Otoplasty and onlay split-rib grafts

### Classification of multiple features

In 1965, Grabb categorized 102 patients into 6 groups defined by varying combinations of skeletal and soft tissue deformities, there were no specific characteristic differences among these 6 groups [18]. Converse et al. provided a classification system for bilateral HFM with four subgroups of 15 patients [15]. Subclassification of groups 1, 2 and 3 was based on a combination of microtia and micrognathia, while patients with severe soft tissue deficiencies and abnormalities of the auricles and facial skeleton belonged in group 4 as the most severe form [16]. In 1977, Edgerton and Marsh divided 17 postsurgical HFM patients into 1 of 4 clinical groups according to the “dominant dysplasia” (mandibular, soft tissue, auricular, or composite) exhibited. The authors suggested that patients with a composite deformity require a treatment plan with a logical sequence for reconstruction with developmental, functional, and psychological considerations [17].

Tenconi and Hall arbitrarily divided 67 patients into 4 major specific phenotypes. Type I was subclassified into classic, microphthalmic, bilateral, and complex types; the other types were limb deficiency, frontonasal and Goldenhar types (Table 6) [28]. However, this system did not include nerve involvement or ear abnormalities, and the described facial underdevelopment was not specific. In addition, the extent of involvement or level of deformity was not designated [23].

**Table 6:** Tenconi and hall classification [28]

Type	Malformations
Type I (A) Classic type (B) Microphthalmic type (C) Bilateral asymmetric type (D) Complex type	Unilateral facial underdevelopment/ without microphthalmos or ocular dermoids Unilateral facial underdevelopment/ with microphthalmos Bilateral facial underdevelopment/ one side of the face is more severely involved Not included in types (A-C)/ not displaying limb deficiency, frontonasal phenotype, or ocular dermoids
Type II Limb deficiency type	Unilateral facial underdevelopment/ with limb deficiency
Type III Frontonasal type	Unilateral facial underdevelopment/ with hypertelorism/ with or without nares separation
Type IV Goldenhar type	Unilateral (type A) or bilateral (type B) facial underdevelopment/ with ocular dermoids/ with or without upper lid coloboma

In 1985, Lauritzen et al. reported an anatomical-surgical classification of HFM with 5 types based on 37 postoperative patients, which was developed by the Toronto Craniofacial Team [20]. Type IA and IB were distinguished by anatomical configuration of the TMJ and amount of orbital involvement, while type through V involved the absence of part of the skeleton with variable extent of sensitivity (Table 7). However, this classification did not take into account nerve involvement or ear abnormalities [23].

**Table 7:** HFM classification by the Toronto craniofacial team [20]

Type	Malformations
Type IA Type IB	Hypoplastic facial skeleton/ horizontal occlusal plane More asymmetric facial skeleton/ tilted occlusal plane
Type II	Absence of the mandibular condyle and part of the ramus
Type III	Absence of the zygomatic arch, glenoid fossa and ramus
Type IV	Partial absence of the zygoma/ Posteriorly and medially displaced lateral orbital wall
Type V	Inferiorly displaced orbits/ with or without anophthalmos

In 1987, Rollnick et al. classified 294 patients with oculoauriculovertebral dysplasia into 5 subgroups according to the presence of microtia, cervical spine anomaly, mandibular hypoplasia, epibulbar dermoids, and lipodermoids, where microtia was the fundamental feature [24].

An alphanumeric coding system was suggested by David et al. based on TNM (tumor, node, metastasis) classification of malignant tumors [56]. Skeletal (S), auricular (A) and soft tissue (T) malformations in 47 patients were independently analyzed with a SAT classification system [16]. The first, second, and third grade of S were adapted from Pruzansky’s classification [14], while the fourth and fifth grade of S were applied to patients with orbital deformations according to Lauritzen et al. (Table 8) [20]. However, this system did not include nerve involvement.

**Table 8:** SAT system by David et al. [16]

Parts	Grades	Malformations
Skeletal (S)	S1 S2 S3 S4 S5	Small mandible with normal shape Distorted but identifiable mandible/ abnormal size and shape Severely malformed mandible/ from poorly identifiable to complete agenesis of ramus S3 + orbital involvement (posterior recession of lateral and inferior orbital rims) S4 + orbital dystopia + hypoplastic and asymmetric neurocranium
Auricle (A)	A0 A1 A2 A3	Normal Small, malformed auricle retaining characteristic features Rudimentary auricle with a hook Malformed lobule with rest of pinna absent
Soft tissue (T)	T1 T2 T3	Minimal contour deficiency/ no cranial nerve involvement Moderate deficiency Major deficiency with obvious facial scoliosis/ severe hypoplasia of cranial nerves, parotid gland, muscles of mastication

In 1991, Vento and colleagues [25] proposed the OMENS classification in 154 HFM patients according to 5 manifestations: mandibular hypoplasia, orbital asymmetry, ear deformity, nerve dysfunction, and soft tissue deficiency (Table 2). Scoring dysmorphic severity on a scale 0–3 was based on conventional 2-dimensional radiographs, photographs, and clinical examination. Orbital asymmetry was assessed by size and position with an arrow indicating displacement direction. Mandibular hypoplasia was classified according to Pruzansky-Kaban classification [8, 57]and ear deformity according to the Meurman [22]. Soft tissue deficiency was graded by modification of the classification by Murray et al. [58]. The OMENS system represents a very accessible, flexible, comprehensive, and largely objective means of classifying the range of abnormalities that make up the spectrum of HFM. The grading systems for each category defines each anatomical anomaly in a very simple and reproducible way covering the full range of dysplastic severity. The use of a numeric classification helps to objectify the many inherently subjective features of this impairment within limitations, and thereby supports the analysis of this population between institutions [54].

The OMENS system has been modified. In 1996, Horgan et al. introduced modified OMENS system with extracraniofacial anomalies, which is called the OMENS Plus system [59]. Because of the frequency of associated macrostomia, which is estimated to occur in 23–35% [60, 61] of the HFM population, Gougoutas et al. [54] modified the original OMENS classification and included both complete and incomplete Tessier no. 7 clefts. The authors also included a field for documenting other miscellaneous anomalies in the OMENS Plus system.

The OMENS system requires further modifications because there is no subgroup defining the degree of malar/midface skeletal deficiency and no subcategorization of minor single-branch paresis [54]. Moreover, it does not include the severity of nerve involvement [49]. Poon et al. suggested that severity be applied in the House-Brackmann facial grading system: 0 = normal nerve function; 1 = mild dysfunction (slight weakness dynamically, eye closure with minimal effort, with normal symmetry and tone at rest), 2 = moderate dysfunction (obvious weakness dynamically, eye closure with maximal effort, but normal symmetry and tone at rest), 3 = severe dysfunction/total paralysis (absent or barely perceptible motion, inability to close eye, with asymmetry at rest) [62].

## Conclusion

This review described various classification systems of HFM, the growth pattern of the maxilla and mandible, and resultant facial deformities. Although the OMENS system classifies HFM's diverse range of abnormalities most comprehensively, there are limitations in that it does not include malar/midface abnormalities and nerve involvement. In the future, based on more clinical studies, it will be necessary to establish a classification system that can address all abnormalities associated with HFM. In HFM patients, the growth of the affected side of the mandible may vary depending on the severity of the mandible deformity and may be less than the growth rate of the unaffected side; however, it is clear that both sides continue to grow during normal growth phases. Therefore, surgical intervention in growing patients should be performed selectively according to the severity of deformity. Understanding growth patterns is important for developing appropriate treatment protocols for correcting asymmetry in adult patients and for minimizing secondary anomalies in growing patients.

## Data Availability

Data sharing is not applicable to this article since no dataset was generated or analyzed during the current study.

## Abbreviations

HFM: Hemifacial microsomia
DO: Distraction osteogenesis
TMJ: Temporomandibular joint
CT: Computed Tomography
